# Measurement of Inhaled Corticosteroid Adherence in Inner-City, Minority Children with Persistent Asthma by Parental Report and Integrated Dose Counter

**DOI:** 10.1155/2012/570850

**Published:** 2012-03-15

**Authors:** Marina Reznik, Philip O. Ozuah

**Affiliations:** Department of Pediatrics, Children's Hospital at Montefiore, Albert Einstein College of Medicine, 3415 Bainbridge Avenue, Rosenthal 4th Floor, Bronx, NY 10467, USA

## Abstract

Parents often overreport adherence to asthma treatment regimens making accurate assessment of medication adherence in clinical practice difficult. This study was conducted to compare two adherence assessment methods clinicians may choose from when assessing patient inhaled corticosteroid (ICS) adherence: parental report and dose counter measurements of metered-dose inhaler (MDI) actuation. Participants included children (*N* = 50) with persistent asthma and their parents (*N* = 50). At enrollment, children received a new, marked ICS at the dose prescribed by their physician. Thirty days following enrollment, we measured ICS adherence by parental report and objectively, with a dose counter. Parental report overestimated ICS adherence when compared to dose counter. We found a statistically significant overall difference between parental report and objectively measured adherence. A dose counter that most ICS inhalers are equipped with may be a more reliable alternative measure of ICS adherence in a clinical practice setting.

## 1. Introduction

Asthma disproportionately burdens low-income African-American and Hispanic children residing in inner cities such as the Bronx, New York [1]. The Bronx, which is predominately Hispanic, is the New York City (NYC) borough with the highest overall rates of asthma hospitalizations, deaths, and prevalence among children and adults [2]. Daily use of inhaled corticosteroid (ICS) medications, the most effective long-term therapy available for patients with persistent asthma, controls symptoms and reduces asthma morbidity [3]. However, adherence to ICS is about 50% for children with asthma [4–7]. Poor adherence to ICS medications contributes to asthma morbidity and has been associated with increased health-care use and decreased treatment effectiveness [3]. Improving adherence in clinical practice setting is difficult because health-care providers do not know if patients are adherent without the use of objective monitoring [8].

Electronic devices attached to inhalers record date and time of medication use and provide objective documentation of adherence [9]. However, these devices are costly [10] and prone to mechanical failure [11, 12], making them impractical for office or clinic practices. In turn, many pediatricians rely on parental report of ICS adherence to guide asthma management in children with persistent asthma [13]. Subjective measures are easily administered and cost-effective, yet often provide overestimated adherence data that may result in unnecessary escalation of treatment [9]. National guidelines recommend that clinicians assess and encourage adherence to recommended therapy during all asthma visits [14]; however, no single successful method of objectively measuring medication adherence in clinical practice has been identified due to a lack of evidence [14].

Recently, several manufacturers have begun to incorporate dose counters into their inhalation delivery devices [15]. Phase III open-label studies in children and adults with asthma and chronic obstructive pulmonary disease (COPD) established clinical functionality, patient satisfaction, and relative accuracy of inhalers with integrated dose counters [15–18]. These studies enrolled patients who were at least 90% compliant with study medication during the screening period [15–18]. There is a lack of literature comparing ICS adherence measured by parental report to that measured by the integrated dose counter in a clinical practice setting. Thus, the objective of our study was to compare two methods of measuring ICS adherence from which clinicians may choose when assessing patient adherence: parental report and dose counter measurements of metered-dose inhaler (MDI) actuation.

## 2. Materials and Methods

We conducted a one-month prospective observational study of 50 low-income, minority children aged 2 to 9 years with physician-diagnosed medically treated persistent asthma who have been prescribed ICS with an integrated dose counter (such as fluticasone propionate hydrofluoroalkane (HFA) inhalation aerosol (Flovent HFA) or fluticasone propionate/salmeterol HFA inhalation aerosol (Advair HFA) and their parents or primary caregivers. Thirty-four (68%) of children have been prescribed Flovent HFA 44 mcg two puffs twice daily, 14 (28%) Flovent HFA 110 mcg two puffs twice daily, 1 (2%) Advair HFA 115/21 2 puffs twice daily, and 1 (2%) Advair HFA 230/21 2 puffs twice daily by their physician prior to the study enrollment. Twenty-five (50%) of children were also prescribed leukotriene receptor antagonist (such as Montelukast (Singulair) 4 mg or 5 mg daily) prior to the study enrollment. Children remained on the prescribed medication regimen, including the dose and preparation, for the duration of the study.

Other study eligibility criteria included: (1) children with at least one acute or same day clinic visit for asthma, Emergency Department (ED) visit, or a hospitalization for asthma in the past 12 months; (2) if the child is 2 years of age at the time of the recruitment, he/she must have at least two prior episodes of wheezing treated and reversible with beta-agonists; (3) primary caregiver speaks English or Spanish; (4) family has a phone. Primary caregiver was defined as an adult who has primary custody of a child and with whom the child spends at least 75% of the week. Although primary caregiver may not be the subject's parent, we used these terms interchangeably. Children with other chronic pulmonary diseases (e.g, cystic fibrosis, bronchopulmonary dysplasia) or presence of tracheostomy were excluded.

Study was approved by the Montefiore Medical Center Institutional Review Board (IRB). Written caregiver consent and child assent were obtained as per IRB guidelines. Families were recruited from two federally qualified Montefiore Comprehensive Health Care Centers located in the poorest section of the Bronx, NY where 27.9% of individuals are below the US poverty level and asthma hospitalization rate is almost twice the rate for NYC [2, 19]. At baseline, we collected sociodemographic information and data about asthma control using current National Asthma Education and Prevention Program Guidelines for the Diagnosis and Management of Asthma [14]. We reviewed child's medical records to determine if additional diagnoses of seasonal allergies and/or eczema have been made by the physician.

All children have been prescribed ICS with an integrated dose counter by their physician prior to study enrollment. At enrollment, children received a new, marked ICS at the physician-prescribed dose. Child's health insurance covered the cost of the ICS for 46/50 subjects. Four subjects had a problem with their health insurance at the time of enrollment, and their ICS was provided by the project. Parents were instructed to administer marked ICS as per physicians' orders (2 puffs twice a day). If used as directed, 120 actuations in the new inhaler suffice for 30 days of treatment. Parents were asked to use only the marked ICS inhaler for the duration of the study. Approximately thirty days after enrollment (range 27–37 days, average 31 days), we visited families at their homes where we administered a survey to measure adherence by parental report and retrieved a marked ICS inhaler that the child received at enrollment.

### 2.1. Measures

#### 2.1.1. Adherence Measured by Parental Report

During the home visit parents were asked the following questions. “During the past 4 weeks, did your child use the marked inhaled corticosteroid pump (controller medication) for his/her asthma?”; “how many puffs and how many times a day did the child use the marked inhaled corticosteroid pump in the past 4 weeks?”; “during the past 4 weeks, how often did your child take marked inhaled corticosteroid (controller medication)? (Answer choices included “every day”, “almost every day”, “several times a week”, “once a week”, and “less than once per week.”)

#### 2.1.2. Adherence Measured Objectively by an Integrated Dose Counter

The integrated dose counter is designed to count downward to zero from the recommended number of actuations and displays the number of actuations remaining in the inhaler. The counter is built into the MDI canister and cannot be reset. Additionally, the device has been engineered to avoid undercounting [15]. We randomly tested 10% of the canisters that we retrieved from the participants (after the data on number of puffs were recorded) and found 0% incidence of the MDI firing but the counter not advancing. To facilitate validity of dose counter data, parents were asked about medication sharing or use of another unmarked ICS at inhaler retrieval. Parents were unaware of the reason for inhaler retrieval.

### 2.2. Statistical Analysis

#### 2.2.1. Definitions

ICS adherence reported by parents was coded as continuous variable: 100% when parents administered ICS to their child “every day”; 75% “almost every day”; 50% “several times a week”; 25% “once a week”; 0% “less than once a week”. Dose counter-measured adherence was calculated as the number of puffs used relative to the number of puffs expected to have been used at 30-day followup.

#### 2.2.2. Data Analysis

Descriptive statistics, including means, ranges, and standard deviations (SD) were calculated for all variables. Wilcoxon signed-rank test, a nonparametric test for paired data, was used to compare the two adherence methods. The Spearman rank correlation coefficient, a nonparametric measure of statistical dependence between two variables, was used to test for association between objectively measured adherence and parental or child age and parental level of education. These nonparametric tests were used because normality was not assumed. All analyses were performed using two-tailed tests with *α* = 0.05. The data were analyzed using SPSS V.19 (SPSS Inc., Chicago, Illinois).

## 3. Results and Discussion

### 3.1. Results

**Figure 1:**
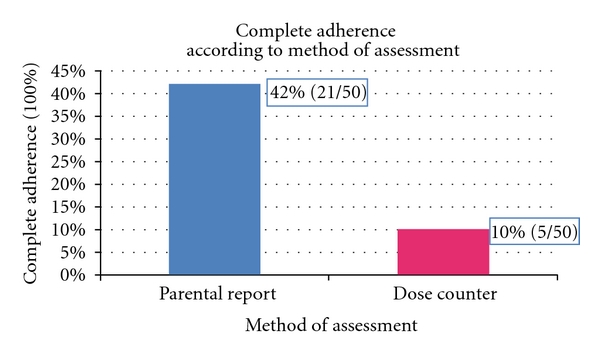
Comparison of two methods of assessment of ICS adherence.

**Figure 2:**
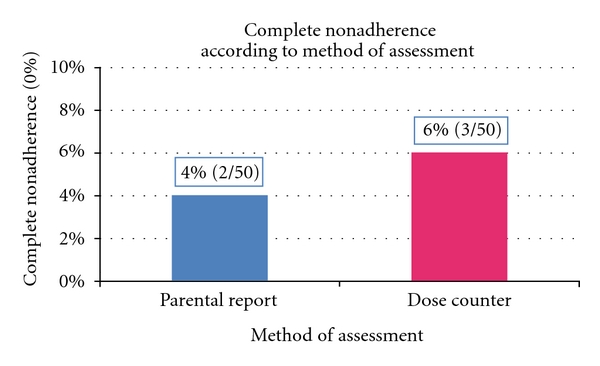
Comparison of two methods of assessment of ICS nonadherence.

A total of 50 children (mean age = 5.6 years (SD 1.9), mean duration of asthma diagnosis 3.9 years (median 4 years)) and their parents (*N* = 50, 96% mothers, mean age = 32.6 (SD 6.9), 64% unemployed) participated.  Table 1 shows sociodemographic characteristics of the study sample. Asthma was not well controlled or very poorly controlled in most children (*N* = 42) (Table 1). Parental report revealed that only 17 (34%) children have ever been evaluated by a pulmonologist (Table 1). Mean ICS adherence as measured by a dose counter was 57.8% (SD 31), (median 60.7%, range 0%–100%). Parental report overestimated complete ICS adherence: 42% (*N* = 21) of parents reported being 100% adherent as compared to 10% (*N* = 5) being 100% adherent as per dose counter (Figure 1). Parental report of nonadherence was accurate: 4% (*N* = 2) of parents reported 0% adherence as compared to 6% (*N* = 3) having 0% adherence as per dose counter (Figure 2). Wilcoxon signed-rank test revealed a statistically significant overall difference between parental report and objectively measured adherence (*P* < .0001). No relationship was found between dose counter-measured adherence and parental or child age (*P* = .732 and *P* = .639, resp.) and parental level of education (*P* = .834). 

### 3.2. Discussion

The impact of poor ICS adherence on asthma treatment outcomes and morbidity has been well documented [3]. The National Heart, Lung, and Blood Institute (NHLBI) Expert Panel Report 3 (EPR-3) recommends that clinicians assess and encourage adherence to medications using parental or self-report [14]. The accuracy of self-report of medication treatment adherence has been examined in many diseases and found to be highly variable and often inaccurate [4, 20, 21]. For example, electronic monitoring revealed that children with asthma used only 50% of prescribed ICS over 6 months, whereas the patients and their parents reported over 80% adherence [4]. Our finding of dose counter-measured ICS adherence of 57.8% supports this earlier report. Moreover, parents in our study overestimated ICS adherence but accurately reported nonadherence.

In addition to self-report, several other adherence assessment methods exist and have been used in clinical trials [8]. These methods differ in the degree of accuracy and objectivity with which patient adherence can be evaluated [8]. However, no single successful method has been recommended for use in clinical practice [14]. As a result, physicians rely on parental or self-report of medication adherence often leading to inappropriate diagnostic and therapeutic decisions [13]. Integrated dose counter has recently been incorporated in several ICS inhalers and found to be reliable [16]. The dose counter not only provides patients with a reliable method for determining the remaining number of puffs, but also may provide clinicians with an objective means of assessing adherence to medication [15]. However, there are no published reports that compared the two adherence methods in a clinical practice setting: adherence measured by parental self-report and adherence measured objectively with a dose counter.

Our study had several limitations. First, our study population was composed of inner-city, minority, poor, and mainly Latino children and parents recruited from two community health centers in the Bronx, NY and may not be representative of all Latino families or minority families in general. However, our study population is representative of the Bronx, NY general population, whose racial distribution in 2010 was about 54% Hispanic or Latino and 36% Black or African American [19]. Second, proportion of children who have been evaluated by an allergist and/or pulmonologist was obtained by parental self-report and does not represent the true number of children who have ever been referred to these specialists by the child's physician but never kept the appointment. This information was not systematically recorded in medical records. Third, integrated dose counters do not provide date and time of MDI use which more expensive, electronic monitors that attach externally to the canister provide [15]. Several studies have documented problems with accuracy of these externally attached electronic devices, including loss of data due to battery drain and recording of nonexistent doses [11, 22–24]. Integrated dose counter is built into the MDI canister and does not require batteries [15].

Evidence from our study indicates that parental report was a nonreliable method for assessing ICS adherence. However, parental report of nonadherence was accurate. Physicians who care for patients with persistent asthma need to have easy-to-use, cost-effective, and objective tool to measure medication adherence in a clinical setting. Integrated dose counter may be such a tool.

## 4. Conclusions

Accuracy of parental report in clinical practice is of concern in light of findings from this study and prior studies revealing that inner-city parents of children with asthma frequently overestimate medication adherence. A dose counter that most ICS inhalers are equipped with may be a more reliable alternative measure of ICS adherence in clinical practice.

## Figures and Tables

**Table 1:** Sociodemographic characteristics of children (*N* = 50).

Characteristic	*N*	%
Gender		
Male	33	66
Female	17	34

Race/ethnicity		
Hispanic^a^	34	68
African American/Black	9	18
Hispanic & African American/Black	6	12
Other race (Indian)	1	2

Asthma control^b^		
Well controlled	8	16
Not well controlled	21	42
Very poorly controlled	21	42

Diagnosed with seasonal allergies^c^	36	72

Diagnosed with eczema^c^	11	22

Have been evaluated by a pulmonologist	17	34

Have been evaluated by an allergist	9	18

Single-parent household	19	38

Parental level of education		
Less than high school	14	28
Graduated high school	15	30
1–3 years of college	15	30
4 years of college or more	6	12

Data in table have been obtained by parental self-report unless noted otherwise.

^a^Hispanic is asked as ethnicity rather than race question as per United States Census Bureau criteria [19].

^b^Asthma control was assessed as per National Asthma Education and Prevention Program Expert Panel Report 3: Guidelines for the Diagnosis and management of Asthma [14].

Well-controlled asthma: Symptoms ≤ 2 days/week; nighttime awakenings ≤1x/month; interference with normal activity—None; short-acting beta_2_-agonist use for symptom control ≤ 2 days/week; exacerbations requiring oral systemic corticosteroids 0-1/year.

Not-well controlled asthma: Symptoms >2 days/week; nighttime awakenings >1x/month; Interference with normal activity—Some limitation; short-acting beta_2_-agonist use for symptom control >2 days/week; exacerbations requiring oral systemic corticosteroids 2-3/year.

Very poorly controlled asthma: Symptoms—throughout the day; nighttime awakenings >1x/week; interference with normal activity—extremely limited; short-acting beta_2_-agonist use for symptom control—several times per day; exacerbations requiring oral systemic corticosteroids >3/year.

^c^Data obtained by medical record review.
